# Implicit learning of convective organization explains precipitation stochasticity

**DOI:** 10.1073/pnas.2216158120

**Published:** 2023-05-08

**Authors:** Sara Shamekh, Kara D. Lamb, Yu Huang, Pierre Gentine

**Affiliations:** ^a^Department of Earth of Environmental Engineering, Columbia University, New York, NY 10027

**Keywords:** organization of convection, machine learning, precipitation extreme, precipitation parameterization, organization metric

## Abstract

Accurate prediction of precipitation intensity is crucial for both human and natural systems, especially in a warming climate more prone to extreme precipitation. Yet, climate models fail to accurately predict precipitation intensity, particularly extremes. One missing piece of information in traditional climate model parameterizations is subgrid-scale cloud structure and organization, which affects precipitation intensity and stochasticity at coarse resolution. Here, using global storm-resolving simulations and machine learning, we show that, by implicitly learning subgrid organization, we can accurately predict precipitation variability and stochasticity with a low-dimensional set of latent variables. Using a neural network to parameterize coarse-grained precipitation, we find that the overall behavior of precipitation is reasonably predictable using large-scale quantities only; however, the neural network cannot predict the variability of precipitation (*R*^2^ ∼ 0.45) and underestimates precipitation extremes. The performance is significantly improved when the network is informed by our organization metric, correctly predicting precipitation extremes and spatial variability (*R*^2^ ∼ 0.9). The organization metric is implicitly learned by training the algorithm on a high-resolution precipitable water field, encoding the degree of subgrid organization. The organization metric shows large hysteresis, emphasizing the role of memory created by subgrid-scale structures. We demonstrate that this organization metric can be predicted as a simple memory process from information available at the previous time steps. These findings stress the role of organization and memory in accurate prediction of precipitation intensity and extremes and the necessity of parameterizing subgrid-scale convective organization in climate models to better project future changes of water cycle and extremes.

## Significance

In nature, precipitation exhibits a significant variance with many extremes of precipitation, while climate models predict a smaller variance in precipitation with a bias toward light rain. Here, using a machine learning approach, we show that 1) we can implicitly learn relevant information about the role of the organization of clouds on precipitation, and 2) including this information can significantly improve precipitation prediction in climate models. Our implicitly learned organization metric explains precipitation stochasticity almost entirely and could replace a stochastic parameterization in climate models. Additionally, this metric exhibits a temporal correlation, caused by the persistence of cloud structure, and thus can be predicted using its own history.

Convection and clouds manifest themselves in various forms and exhibit multiscale structures, ranging from being randomly distributed to being highly organized (i.e., clustered). This organization, which can persist from a few hours to several days, can modify atmospheric moisture distribution, radiation, and precipitation intensity (1, 2). In a random, unorganized, convecting atmosphere, moisture has a narrow distribution. Organization increases the variance of the moisture distribution by increasing the humidity in the clouds’ surrounding areas and by drying out more distant regions.

Nevertheless, this reduction in moisture does not necessarily correspond to a decrease in precipitation intensity, as predicted by some precipitation schemes (3). Organized convection creates a locally humid environment that promotes longer-lasting, stronger, and more humid convective clouds; because the air entrained at the edge of convection is moister, it does not substantially reduce the buoyancy of convection. Furthermore, organization may generate a self-reinforcing moisture memory, increasing the chances of convective precipitation in regions that have experienced deep convection and precipitation recently (4). The degree of organization can also affect the precipitation efficiency (5), defined as the ratio of surface precipitation to cloud condensate, so that the inclusion of an organization metric in climate models might be important for more realistic predictions of precipitation extremes in a warming climate (6). The organization of clouds also affects intercloud interactions and cold pool properties. Convection triggered by the collision of cold pools (namely, areas of relatively low temperature generated by the evaporation of rain or melting of ice) is more likely to result in extreme precipitation (7).

The above-mentioned processes emphasize the crucial role of convective organization in modifying the distribution of moisture and precipitation. However, for climate models that solve discrete forms of continuous equations of motion at a coarse resolution of about 100 km, convection is spatially too small to be resolved. As a result, its impacts on resolved variables are approximated as a function of these variables, a process known as parameterization. Convective parameterizations typically do not represent subgrid-scale organization. This approximation raises the question of whether we can rely solely on resolved variables for the representation of convection in parameterizations or whether we need to consider the smaller-scale structure of convection as well.

Typical mass-flux convection schemes, used in general circulation models (GCMs), employ a model of convective updrafts, under the assumption of quasi-equilibrium (QE) (8). These parameterizations do not include any organization or interaction among convective updrafts, treating them as randomly distributed convective plumes. Cloud cover parameterization and their impact on radiation sometimes use some representations of organization, but these remain largely ad hoc (9). This raises the question as to whether we need to incorporate some information about subgrid-scale organization to better model precipitation intensity and its distribution in GCMs.

**Fig. 1.**
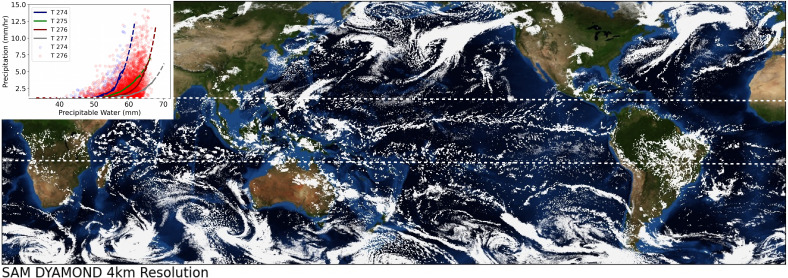
Global storm resolving model. Snapshot of a cloud scene on 24 February 2016 from SAM as part of the DYAMOND dataset. Ten days, randomly selected, of the tropical regions (displayed between the two white dashed lines) from this simulation are used for this analysis. The inset plot shows precipitation versus precipitable water for 10 d of SAM simulations. Lines show the precipitation conditionally averaged by 0.3-mm bins of precipitable water and for 1-K bins of free tropospheric temperature. Scatter dots show the spread in precipitation for each bin of precipitable water and averaged free tropospheric temperature across the simulation domain and time period.

GCMs’ prediction of the global mean precipitation, which is controlled by the energy balance of the atmosphere, is typically close to the observed value (10). Additionally, precipitation averaged over bins of precipitable water (PW), which represents the total amount of water vapor in a column of the atmosphere, exhibits a simple power-law behavior (11) with respect to PW, increasing rapidly as PW increases. However, precipitation shows large variability within each bin (Fig. 1). In other words, conditioned on large-scale quantities, precipitation exhibits strong stochasticity. Many climate models have shortcomings in accurately reproducing precipitation statistics, and they sometimes rain too often and too little (the so-called “drizzle problem”). One approach to address this shortcoming is by adding noise to the convective tendencies predicted by the parameterization (12, 13). This stochastic parameterization has shown promise to improve weather and climate model prediction of precipitation statistics and reduce its biases (14), yet this noise is not directly connected to any physical processes and thus is challenging to parameterize. Subgrid-scale organization (at scales ∼100 km) is one source of stochasticity regulating precipitation intensity at the climate scale (15). The failure of traditional deterministic parameterizations to accurately reproduce the statistical behavior of precipitation could potentially be rooted in their neglect of subgrid-scale organization.

Parameterizing subgrid-scale organization has also previously been suggested as a potential approach to circumvent the so-called “entrainment” problem. Mapes and Neale (16) included a prognostic and empirical “organization” variable in their convective parameterization, with the effect that subgrid-scale structure led to deeper and stronger convection than in the unorganized case. Their prognostic “organization” parameter was a single empirical dimensionless scalar, tuned such that it had a steady-state value on the order of unity and a timescale on the order of a few hours.

One obstacle in integrating subgrid-scale organization into parameterizations is determining how to obtain useful information from these subgrid structures that can aid in approximating unresolved variables such as precipitation, convective mass flux, or radiation, which may be reliant on such an organization. It is essential to not only comprehend how organization influences various processes but also precisely measure its overall impact at the GCM scale, so as to incorporate it into the parameterization. To address this challenge, we employ machine learning to implicitly learn subgrid-scale organization and develop a precipitation parameterization that incorporates these data-driven organization metrics. Machine learning techniques such as deep neural networks provide a powerful opportunity for developing new parameterizations, given the availability of high-resolution simulations of the atmosphere. Neural networks (NNs) can closely approximate the underlying function that relates observed quantities (or inputs) to the target quantities. Machine learning has recently proven powerful for parameterizing subgrid-scale processes in climate models, such as convection and precipitation; by training neural networks to emulate higher-resolution simulations averaged at coarse resolution, as in those high-resolution simulations, some processes such as convection are explicitly resolved (17–21). Neural networks can emulate unresolved processes from resolved variables, as they can capture complex nonlinear behavior of a physical system, given that they have access to the necessary predictors and thus can be used for hypothesis testing by evaluating the influence or relevance of specific inputs to a particular prediction (in our case of precipitation at the coarse scale). Machine learning has also shown tremendous skill in analyzing images and their complex spatial structure and thus might be well suited to investigate 2D organization and as a hypothesis tool to demonstrate the importance of subgrid information to best reproduce precipitation. Here, we take advantage of this capacity of NNs to test two hypotheses: namely, to

- evaluate to what extent coarse-scale precipitation (∼100 km) is predictable using only coarse-scale quantities, and
- investigate whether informing the neural network with subgrid-scale organization information improves its prediction of precipitation.

The data that we use for training the neural networks are the high-resolution simulations produced by the System for Atmospheric Modeling (SAM) (22), which was run as a part of the DYnamics of the Atmosphere general circulation Modeled on Nonhydrostatic Domains (DYAMOND) Phase 2 Intercomparison Project (23) and which are publicly available. The original resolution of SAM-DYAMOND is 4.2 km, and the two-dimensional quantities are written every 15 min. We discuss the realism of the DYAMOND dataset, specifically its precipitation product, in more detail in the methodology section.

**Fig. 2.**
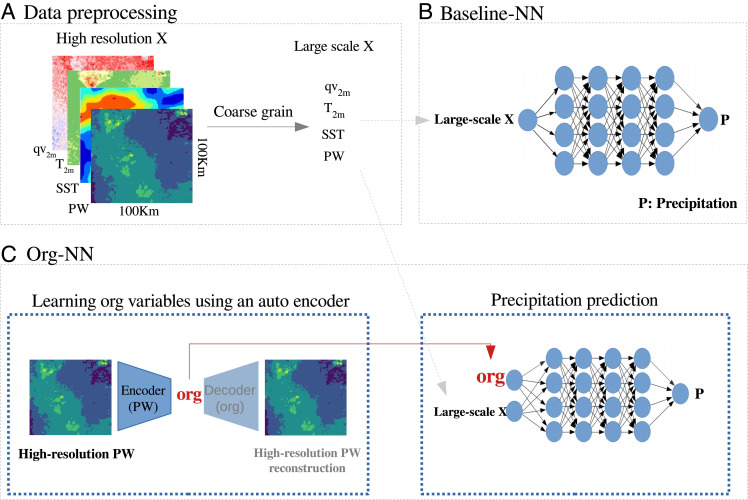
Overview of proposed framework for parameterizing precipitation. (*A*) Coarse-graining the high-resolution data. (*B*) Baseline-NN architecture: This network receives coarse-scale variables (e.g., SST and PW) as input and predicts coarse-scale precipitation. (*C*). Org-NN architecture: The *Left* panel shows the autoencoder that receives the high-resolution PW as input and reconstructs it after passing it through a bottleneck. The *Right* panel shows the neural network that predicts coarse-scale precipitation. The input to this network is the coarse-scale variables (as for the baseline network) as well as *org* extracted from the autoencoder. The two blocks are trained simultaneously.

To prepare the data for training a neural network, we coarse-grain two-dimensional variables of SAM-DYAMOND by horizontally averaging to typical climate model resolution (for instance, 100 km), as shown in Fig. 2*A* and discussed in the method section in more detail. We refer to these averaged variables as coarse-scale. Furthermore, we discard all nonprecipitating and land pixels, thus to narrow the focus of this study down to predicting precipitation intensity over the tropical ocean (20°S - 20°N). This choice reduces the heterogeneity of the feature space and allows us to build a minimal but robust model with a significantly smaller number of inputs compared to previous studies (17, 18, 20, 21). In *SI Appendix*, we discuss how including land modifies the results.

Our baseline neural network is a vanilla, fully connected feed-forward network (Fig. 2*B*) that receives coarse-scale variables as input and predicts coarse-scale precipitation. The inputs are PW, sea surface temperature (SST), near-surface specific humidity (qv2m), and near-surface air temperature (T2m), and the output is precipitation. In simple precipitation models, SST and PW are commonly used as predictors of precipitation intensity (11, 24). Muller and O’Gorman (25) show in their cloud-resolving model that the boundary layer water vapor sets a stability threshold that controls the onset of deep convection. Following their finding, we use qv2m as a proxy of boundary layer water vapor, as we find a strong correlation between these two variables (*SI Appendix*, Fig. S1*A*). We also use T2m as a predictor because of its high correlation with temperature averaged over the boundary layer (above 850 hPa), as shown in *SI Appendix*, Fig. S1*B*. From qv2m and T2m, the network can potentially learn the relative humidity of the boundary layer, an important factor in the evaporation of rain and thus precipitation efficiency, which largely affects precipitation intensity (5, 26).

Thus, most results are based on this set of inputs coarse-grained over a block of 32 × 32 (or 130 × 130 km^2^). Nevertheless, we explore several other choices of inputs and coarse-graining that we describe in *Methods* and discuss their impact on the results when relevant.

## Results

### Predicting Precipitation from Coarse-Scale Quantities.

To investigate the first hypothesis, i.e., the predictability of precipitation using coarse-scale quantities only, we use a neural network depicted in Fig. 2*B*, which we refer to as baseline-NN.

**Fig. 3.**
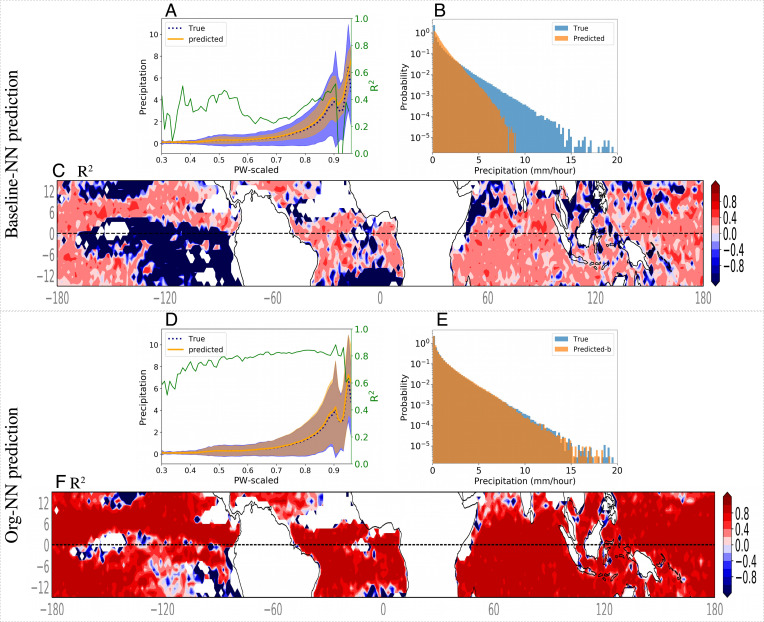
Performance of the NN. (*A*–*C*) trained using only coarse-scale variables and (*D*–*F*) trained using coarse-scale variables as well as *org* metric as input. *A* shows true (blue) and predicted (orange) coarse-scale precipitation averaged over PW bins (1 mm) for training using only PW, SST, qv2m, and T2m. Shading shows the SD of coarse-scale precipitation for each bin of PW. The green line plots the *R*^2^ across PW bins. Panel *B* shows the probability density function (pdf) of precipitation for true (blue) and prediction from panel a (orange). Panel *C* displays the *R*^2^ computed for each latitude and longitude location across time steps for panel *A*. Panels *D*–*F* show the same as *A*–*C* but predicted as by Org-NN, which includes the *org* metric in its inputs.

Fig. 3*A* shows the precipitation predictability when the NN uses as input coarse-scale PW, SST, qv2m, and T2m. The latter two variables inform the baseline-NN about the boundary-layer condition (27). To construct this plot, we bin coarse-scale PW, then average coarse-scale precipitation, predicted and true, over each bin of PW. We also compute the variance of coarse-scale precipitation values that fall within each bin of PW. The plot shows the bin-averaged precipitation (orange line and blue dashed line) and the variance within each bin (shading). The baseline-NN accurately recovers the critical behavior of mean (bin averaged) precipitation conditioned on PW and its rapid transition past a critical point. However, it cannot explain the precipitation variability observed in the global storm resolving simulations, and its performance, measured by *R*^2^ across all samples, is about 0.45. The low *R*^2^ reveals that even though the baseline-NN captures some of the variability in precipitation, it does not find a strong relationship between inputs and precipitation. Furthermore, *R*^2^ computed at each bin of PW (Fig. 3*A* green line) does not exceed 0.5.

Comparing the probability density function (pdf) of precipitation predicted by the baseline-NN with the true precipitation (Fig. 3*B*) reveals that the model also fails to predict the tail of the distribution, in line with climate models’ prediction of precipitation and their difficulty in representing extremes.

To verify the importance of surface fluxes on the prediction of precipitation, we run an extra test in which we include coarse-scale surface-sensible and heat fluxes alongside the four variables mentioned above in the input of the baseline network. This inclusion marginally improves overall *R*^2^ by 0.05∼0.1. This improvement mostly occurs at locations with large PW for which precipitation intensity is small, potentially related to the diurnal cycle of precipitation; yet, the network still underestimates extreme precipitation (*SI Appendix*, Fig. S2*A*).

We run further tests with our baseline-NN, in which we also include total cloud cover (at the coarse scale) as an input of the network. In climate models, total cloud cover is a parameterized variable and not directly related to precipitation, so that including it as input of the NN could provide hints about the condensed water, which is directly used for the parameterization of precipitation. This inclusion (*SI Appendix*, Fig. S2*B*) improves the prediction negligibly but emphasizes that the mean cloud cover does not provide relevant information required for accurate prediction of precipitation. Additionally, further analysis confirms that including Convective Available Potential Energy (CAPE) or Convective INhibition (CIN) as predictors does not improve the prediction (*SI Appendix*, Fig. S2*C*).

To summarize, the baseline-NN has a low skill in accurately predicting precipitation and its variability, even though the overall behavior of precipitation, averaged over bins of PW, is decently predictable from coarse-scale quantities (PW, SST, and boundary layer information). In this work, we use only the near-surface and column-integrated values as input of the NN; thus, we cannot rule out that including the whole column profile may improve the prediction. Yet, based on previous studies, even including the whole column of the atmosphere as input to a machine learning model did not fully capture the stochasticity observed in GCM-scale precipitation, as shown in previous work (21). Colin et al. (28), using small-domain cloud-resolving simulations, showed that homogenizing the moisture field horizontally while keeping the domain mean constant significantly affected precipitation. Furthermore, Yuval and O’Gorman (20) indicated that precipitation predictability is significantly reduced by coarsening the resolution, despite the use of moisture, temperature, and wind at all the vertical levels, from the surface to the free troposphere. This indicates that either relevant information for an accurate coarse-scale prediction of precipitation is missing or that the precipitation is stochastic and thus that its actual value is largely unpredictable. In the next section, we investigate the next hypothesis, which is precipitation variability is explainable by the inclusion of an organization metric.

### Org Informed Prediction of Precipitation.

Our hypothesis for the failure of the baseline-NN to predict precipitation variability is that it is due to the lack of information on subgrid-scale variability, which is not present in coarse-scale variables, such that additional subgrid-scale information needs to be included in the inputs of the NN. Here, we specifically investigate the potential importance of subgrid-scale cloud patterns, i.e organization, and whether including this information in the neural network can improve its prediction. Over the last three decades, many studies have investigated the impact of organization on the dynamics and thermodynamics of convection as well as on precipitation. In parallel, more than 20 different metrics have been developed to quantify the degree of organization or, more generally, the cloud pattern (29). *SI Appendix*, Fig. S3 shows the correlation between the time series of different metrics applied to the DYAMOND dataset. These metrics capture some aspects of the organization and can potentially be used to inform the coarse-scale variables about subgrid-scale cloud patterns for predicting precipitation. However, these metrics have been designed for large domains (> 200 km), while for climate-size domains (100 to 200 km) where convection might cluster to some degree, these metrics usually fail to provide valuable information. Furthermore, each of these metrics targets a specific aspect of cloud organization that may not be directly relevant to predicting precipitation.

We instead turn this approach on its head and try to learn an implicit representation of organization that is most relevant to precipitation prediction. To do so, we extract information relevant to predicting precipitation from a high-resolution field using a dimensionality reduction technique known as an autoencoder. An autoencoder (AE) is a powerful nonlinear dimensionality reduction approach that has been originally developed for image processing (30). The encoder part of the AE nonlinearly projects high-resolution inputs into a low-dimension nonlinear manifold that efficiently describes the data. This low-dimension representation, i.e., a latent space, embeds optimal information needed from the high-resolution field to reconstruct precipitation. Here, we apply this AE to high-resolution PW and extract its latent representation (hereafter named *org*) to inform our precipitation-predicting network about the organization. By coupling the AE with the NN, which predicts precipitation, and training the two networks in parallel, the AE directly receives feedback from the objective function of the NN through back-propagation (the process of minimizing the loss function by adjusting the weights and biases of the NN). Thus, the AE is forced to extract relevant information that improves the prediction of precipitation. Fig. 2*C* shows the architecture of our network. We refer to this network as Org-NN. Importantly, we impose rotation invariance of the loss function measuring the organization of the high-resolution field, as the organization of clouds should not depend on its exact location, nor on the rotation of the field (*Methods*). The *Left* block of the schematic (Fig. 2) shows the autoencoder that receives high-resolution PW anomaly as input. The *Right* block shows the fully connected feed-forward NN, with the same number of hidden layers and neurons as baseline-NN. This network receives the coarse-scale variables along with *org* and predicts precipitation. These two blocks are trained simultaneously. In other words, we train two networks end to end in parallel: one that reduces the dimension of the high-resolution field into a few latent variables—the *org*—and the other one that predicts precipitation with SST, PW, qv2m, and T2m (as previously) with the addition of organization latent variables *org*. The dimension of *org* is set to 4. Further tests with different *org* dimensions are discussed in the Methods section and summarized in *SI Appendix*, Table S2 and Fig. S5.

Fig. 3*D*–*F* shows the Org-NN prediction of precipitation. Org-NN demonstrates a significant improvement as compared to the baseline-NN. The *R*^2^ of this prediction increases to 0.9 when computed across all data points. Reducing the number of org variables to 2 or changing the resolution does not significantly change the results as summarized in *SI Appendix*, Table S2 and Fig. S5. *R*^2^, computed for each bin of PW, is close to 0.80 for almost all bins except where precipitation is small (e.g., scaled-PW ∼ 0.3).

We further quantify the Org-NN performance by comparing its probability density function (PDF) with the one of the true precipitation from the storm-resolving model (Fig. 3*E*). Org-NN fully captures the PDF, including the tail of the distribution, which corresponds to the precipitation extremes. Additionally, Fig. 3*F* shows the *R*^2^ of the Org-NN computed for each latitude-longitude grid across time steps. The white patches in this figure have precipitation smaller than 0.05 mm/h, so they are excluded from the input of the model. Org-NN has significantly larger *R*^2^ (> 0.8) except for regions that are close to the points which do not have precipitation larger than the threshold.

In climate models, the modeling and representation of hourly and subhourly precipitation extremes, which are dominated by deep convective precipitation, continue to be one substantial source of uncertainty. Our results demonstrate a significant improvement in precipitation prediction with the inclusion of *org*, suggesting that the subgrid-scale structure is potentially an important piece of information currently missing in the parameterization of convection and precipitation in climate models.

### Prediction of the Org Variables.

Including *org* variables in the input of the neural network significantly improves the prediction of precipitation; however, in climate models, where high-resolution fields are not available to be used for the prediction of *org*, how can *org* be generated? One approach to address this issue would be to predict *org* from coarse-scale variables resolved by a climate model. Rephrased differently, can we apply a diagnostic model using coarse-scale variables as input to predict *org*? Additionally, from observations, we know that organization can persist over time, thus exhibiting a strong temporal memory, meaning that *org* variables should carry some information that persist over time and could be advected, potentially with the mean field. Mapes and Neale (16) integrate this idea in their *org* metric by making it time-dependent and advective. We adopt this approach and include large-scale variables and *org* at the current and previous time steps to predict the *org* at the current time. Mathematically this is expressed as

[1] $$\begin{array}{c} \begin{array}{c} org\left(t\right)=F[\bar{X}\left(t-n_{\mathrm{LS}}\Delta t,...,x-\Delta t,t\right),\\ org(t-n_{\mathrm{org}}\Delta t,...,t-\Delta t),\theta ]\\ (n_{\mathrm{LS}}=0,2,4;n_{\mathrm{org}}=0,1,2), \end{array} \end{array}$$

where ℱ is a function approximated by a fully connected feed-forward neural network, $\bar{X}$ is the coarse-scale variables (SST, PW, qv2m, T2m, and surface fluxes), and *θ* is the neural network parameters. *Δt* is the time resolution of the data and equals 15 min for the DYAMOND data. *n*_*LS*_ and *n*_*org*_ represent the maximum lagged time step of large-scale variables and the *org* variables that we include as the input features. Thus, the network that approximates ℱ has two sets of inputs, the coarse-scale variables from current and up to *n*_*LS*_ previous time steps and the *org* variables of up to *n*_*org*_ prior time steps. Additionally, we also build neural networks to predict *org* using only *org*’s previous time steps without any coarse-scale input. In this case, the first ($\bar{X}$) term in Eq. **1** is no longer retained. All inputs are at the same location as predicted org; that is, the model is local. With this model, we design two sets of tests: 1) including only the coarse-scale variables (*n*_*org*_ = 0) as input to verify the predictability of *org* from historical information at coarse-scale, and 2) including both coarse-scale and *org* history as input to explore the predictability of *org* from its own history.

**Fig. 4.**
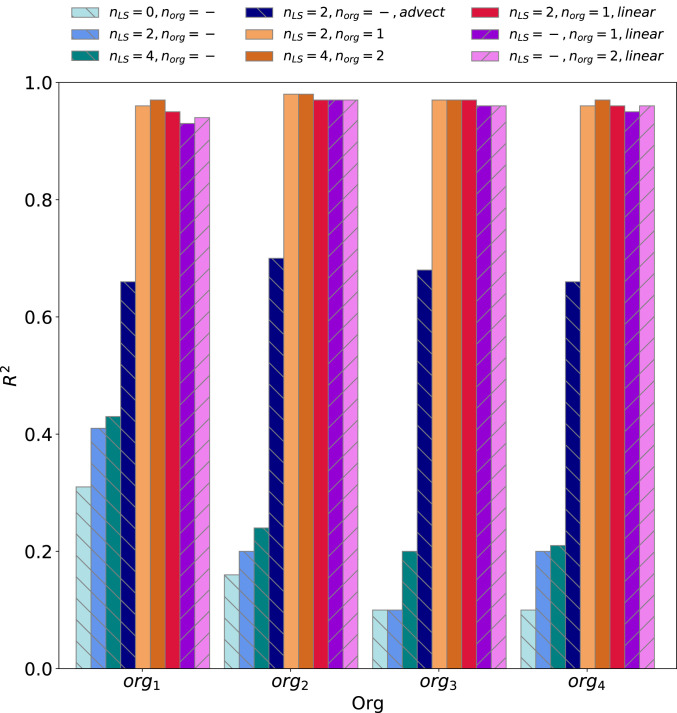
The figure summarizes *R*^2^ of *org* prediction (Eq. **1**). The neural networks that use only coarse-scale variables as input are marked by back slashes; the neural networks that use only previous *org* values as input are marked by forward slashes. For the neural networks with both large-scale variables and previous *org* as input, *n*_*LS*_ and *n*_*org*_, respectively, represent the prior time steps of coarse-scale variables and org history included as the input to the neural network (Eq. **1**). For instance, *n*_*org*_ = 1 and *n*_*LS*_ = 2 means using one prior time step of *org* and current and two previous time steps of coarse-scale variables as input to the neural network ℱ (Eq. **1**). The test “advect” includes neighboring coarse-scale pixels in the inputs, and the tests ‘*linear*’ use a multiregression model instead of the neural network.

**org* prediction with coarse-scale input:* We run three tests with *n*_*LS*_= 0, 2, and 4, which, respectively, correspond to using only the current time step, the current and two previous time steps, and the current and four previous time steps. Fig. 4 summarizes the *R*^2^ of these tests. Predicting org with $\bar{X(t)}$ (i.e., *n*_*LS*_ = 0) has *R*^2^ ∼ 0.3 for the first org variable and *R*^2^ ∼ 0.1 for three others (Fig. 4). Including 2 previous time steps (i.e., *n*_*LS*_ = 2) increases *R*^2^ of all *org* predictions by about 0.1, while increasing *n*_*LS*_ to 4 results in a minor increase in *R*^2^ (Fig. 4 and *SI Appendix*, Fig. S4) compared to *n*_*LS*_= 2. This finding indicates that the *org* variables, particularly *org*_1_, are to some extent predictable from historical information of coarse-scale variables with the best results achieved with *n*_*LS*_= 4 (1 h). Thus, large parts of information encoded in *org* are not retrievable from the historical (and local) coarse-scale variables.

In the three tests mentioned above, we use only coarse-scale information of the same location for which we predict *org*, thus ignoring the spatial advection of *org*. In addition to temporal correlation, *org* can also be spatially correlated. Moreover, *org* encodes not only the persisting subgrid-scale structures but might also capture the coarse-scale front or waves propagating and affecting the moisture field. To take account of this source of *org*, as well as the spatial correlation, we modify the inputs to ℱ to include the coarse-scale variables of neighboring pixels as well. For this specific test, then, we set *n*_*LS*_ = 2, *n*_*org*_ = 0, and $\bar{X}$ is a 3x3 horizontal block for predicting *org* at the center of this block. This test, shown in dark blue color in Fig. 4, has *R*^2^ ∼ 0.66 − 0.70, which is significantly larger than the test with similar historical coarse-scale data but only local information. The larger predictability of *org*, when the neighboring coarse-scale variables are known, suggests that *org* probably captures more than just persistent subgrid-scale structure and encodes propagating coarse-scale flow, which influence moisture field and precipitation.

*Prediction with coarse-scale and historical org inputs:* As our second set of tests, we include *org* history, alongside the large-scale variables, in the input of the neural network ℱ. With this setup, we perform a test with *n*_*LS*_ = 2 and *n*_*org*_ = 1, which corresponds to including coarse-scale variables of current and two previous time steps and *org* of one previous time step, and a second test with *n*_*LS*_ = 4 and *n*_*org*_ = 2. We find that making *org*(*t*) dependent on its own history improves the prediction of *org*(*t*) significantly (Fig. 4). The test with *n*_*LS*_ = 2 and *n*_*org*_ = 1 has *R*^2^ > 0.95 for all four *org* variables. This indicates the substantial predictability of *org*(*t*) given *org*’s history, due in part to the persistence of subgrid-scale structures over a period larger than the 15-min time step of our data.

Given the significant temporal correlation among *org* variables, one can simply predict *org*(*t*) using an autoregressive or multiregression model. To do so, we replace the neural network, ℱ, with a multiple linear regression and predict *org*(*t*) as a linear function of *org*(*t* − *dt*) and $\bar{X}$ of current and previous time steps, as summarized in Fig. 4, while keeping the model local (i.e., no neighboring pixels). The result, shown in Fig. 4), suggests that once the previous *org* value is known, *org*(*t*) becomes highly predictable even with only one prior *org* time step. For a specific test where we exclude coarse-scale variables from the input and set *n*_*org*_ = 1 (thus reducing the multiregression model to an autoregressive model), *R*^2^ is higher than 0.95 for all *org* values. This approach would then be preferable, compared to the others mentioned above, due to its simplicity and the need for less historical data for its input, which is crucially advantageous for the implementation in a GCM.

### What Does org Measure?.

Previous *org* metrics have been developed using statistical and geometric arguments (29), but here, we have relied on a data-driven approach to develop an optimal metric for organization that is specifically targeting the predictability of precipitation at the grid scale. Because neural networks have nonlinear activation functions between layers, they are more challenging to interpret than linear models. To understand what the encoder is learning, we additionally train a decoder to reproduce the high-resolution fields from the latent representations. This decoder uses a rotation-invariant loss function (31); this imposes a constraint such that high-resolution fields that are rotations of one another (by *nπ*/2, where *n* is a random integer number) are mapped to the same latent representation, as rotations should not be physically meaningful for predictions of precipitation.

**Fig. 5.**
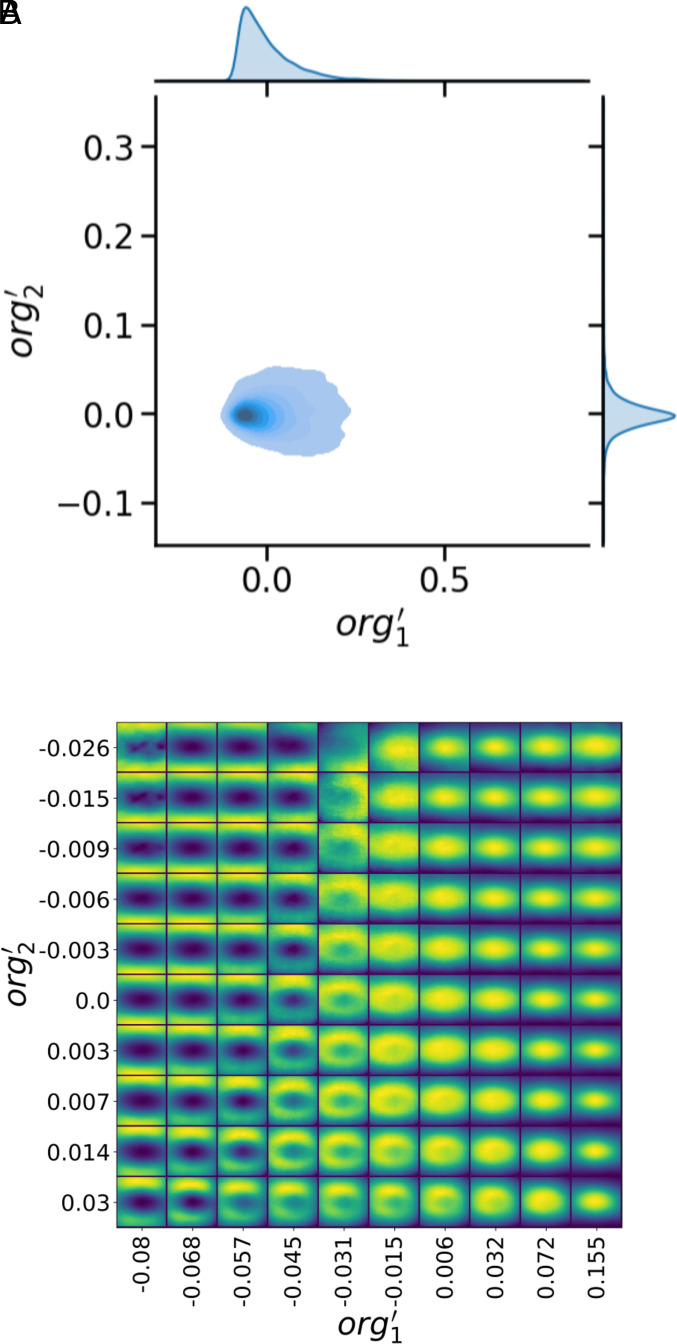
Latent space of *org*. (*A*) Joint probability distribution of the first two principal components of the latent variables for the *d*_*org*_ = 2 case (denoted as $org_{1}\prime$ and $org_{2}\prime$) from predictions of the encoder trained on the DYAMOND simulations. The marginal distribution are shown on the *Top* (for $org_{1}\prime$) and *Right* side of the plot (for $org_{2}\prime$). (*B*) Visualization of the latent representation for the *d*_*org*_ = 2 case. Color scaling is relative to the minimum and maximum values for PW in each reconstructed high-resolution field to best show small-scale contrast. Values for the $org_{1}\prime$ and $org_{2}\prime$ are chosen to be the midpoint of the deciles of their marginal distributions. *SI Appendix*, Fig. S7 shows the same figure but with the color scale relative to the absolute contrast across all the reconstructed high-resolution fields.

To directly visualize the latent space, we first determine the observed distributions of the *org* parameters learned from the simulation data using the encoder. We consider cases where the latent space has a dimension of *d*_*org*_ = 2 and *d*_*org*_ = 4 (at a single time step). We denote these latent variables as *org*_1_ and *org*_2_ for *d*_*org*_ = 2, and *org*_1_, *org*_2_, *org*_3_, and *org*_4_ for *d*_*org*_ = 4. For both the *d*_*org*_ = 2 and *d*_*org*_ = 4 cases, we find that the learned parameters are highly correlated with one another (joint distributions in *SI Appendix*, Figs. S6*A* and S8). We apply principal component analysis to the *org* variables to linearly transform these variables to a coordinate system where the axes lie along the directions of greatest variance of the dataset. The joint and marginal distributions for the first two principal components for the *d*_*org*_ = 2 case (denoted as $org_{1}\prime$ and $org_{2}\prime$) are shown in Fig. 5*A*, and for the first four principal components for the *d*_*org*_ = 4 case are shown in *SI Appendix*, Fig. S9.

To effectively map out the latent space across the range of *org* parameters observed in the DYAMOND simulations, we then use the trained decoder to reconstruct the high-resolution fields sampled across the distribution of values observed in the training dataset along the directions of greatest variance for the latent variables. The values of *org*_1_ and *org*_2_ that are input to the decoder to produce a reconstruction of the high-resolution fields characteristic of that portion of the latent space are determined by finding the average values for each decile of the distributions of the transformed variables ($org_{1}\prime$ and $org_{2}\prime$). Fig. 5*B* shows the latent space visualization for the *d*_*org*_ = 2 case. We similarly visualize the latent space along the first two principal components for the *d*_*org*_ = 4 case in *SI Appendix*, Fig. S10, and for the next leading principal components in *SI Appendix*, Fig. S11; in this case, we fix the values for the other two principal components to the central values of their marginal distributions.

Mapping out the latent representations along the first two principal components of *org* for the *d*_*org*_ = 2 and *d*_*org*_ = 4 cases indicates that the learned *org* variables capture information about how aggregated the high-resolution precipitable water fields are. The first principal component (which accounts for 94.8% and 98.2% of the explained variance for the *d*_*org*_ = 2 and *d*_*org*_ = 4 cases, respectively) in particular demonstrates increasing aggregation moving from *Left* to *Right* in Fig. 5*B*. Moreover, these two main modes of variability are robust to the number of *org* parameters in the latent space, as the latent spaces for both the *d*_*org*_ = 2 and *d*_*org*_ = 4 case are very similar for these first two principal components (Compare Fig. 5*B* and *SI Appendix*, Fig. S10). In addition, examining the absolute scaling of precipitable water in the latent space (*SI Appendix*, Fig. S7) indicates that $org_{1}\prime$ is not simply correlated to the mean value of precipitable water but is instead a measure of the degree of aggregation of the high-resolution field.

## Discussion

Whether and how to incorporate the subgrid-scale structure into a climate model parameterization has been a persistent question in the parameterization of convection. It is also relevant to many other problems in climate science where the overall impact of a complex field has to be approximated, such as ocean surface temperature anomalies or the heat conductivity of sea ice. In this work, we suggest an elegant approach to implicitly learn the subgrid-scale structure and effectively incorporate this into a parameterization. Our approach models the two scales by two different networks: the unresolved scales using an autoencoder and resolved scales using a feed-forward neural network.

The results presented here suggest that coarse-scale variables (at a scale of climate models ∼100 km) are not sufficient predictors for accurate replication of precipitation statistics because of the lack of inclusion of subgrid organization. This finding calls into question current climate model parameterizations of convection and precipitation that ignore any degree and mode of organization at the subgrid scale. The current climate model representation of convection is typically based on an ensemble of unorganized, randomly distributed buoyant updrafts, without any interaction or memory of the previous state of the system. This deficiency has been suggested as one major reason for the well-known climate model bias toward light rain (7, 16, 32), i.e., the so-called drizzle problem.

We have shown here that including convective organization subgrid-scale information (in a neural network) significantly improves the prediction skill of precipitation, particularly the stochasticity and extremes of precipitation—a long-lasting issue for climate models. This finding suggests that precipitation stochasticity at the climate model scale is linked to subgrid-scale structure and can be fully explained when this information is incorporated into the parameterization.

Our data-driven organization variables, *org*, extracted from a high-resolution field of PW were shown to carry the required information needed to accurately predict precipitation at the coarse scale. In addition, these organization metrics were shown to be predictable as a memory process informed by historical coarse-scale variables or *org* of a previous time step. The studies of self-aggregation using small-domain cloud-resolving models have emphasized the importance of memory for the persistence of organization. In that case, the memory has been attributed to moisture–convection feedback, moisture–radiation feedback, etc. (2, 4, 28). Although we have not investigated the source of memory, our results suggest that historical coarse-scale variables are informative of the *org* variable to some degree. More importantly, the strong temporal correlation between *org* variables indicates that it may be related to persistent subgrid-scale structures, and thus, these structures are important for precipitation. An important avenue for future research is exploring the physical processes and causal mechanisms that give rise to this memory.

Our data-driven parameterization of subgrid-scale structure can provide guidance for existing parameterizations by learning the distribution of *org* variables and the temporal and spatial correlations between them. This distribution can then be used to inform the noise term in existing stochastic parameterizations. Additionally, it is important to investigate the relevance of subgrid-scale structure in predicting convective tendencies and radiative cooling of the atmosphere to further improve data-driven parameterizations. Since these parameterizations are all connected, it is crucial to investigate whether a common set of *org* variables can be learned and used effectively across all parameterizations where the subgrid-scale structure might have an impact.

Our finding helps to guide and improve the representation of organization in climate models, one of the grand challenges in “Clouds, Circulation and Climate Sensitivity” (33). We provide a pragmatic way to tackle two problems at once: the representation of cloud organization and precipitation stochasticity in coarse-scale models. We also emphasize the key role of organization and its predictability on precipitation prediction.

## Materials and Methods

### Data.

The dataset was produced by the System for Atmospheric Modeling (SAM) (22), as a part of the DYnamics of the Atmosphere general circulation Modeled on Nonhydrostatic Domains (DYAMOND) Phase 2 Intercomparison Project of global storm-resolving models (23).

The precipitation from DYAMOND simulations spatiotemporally averaged over the tropics is comparable to the observation (23). For instance, SAM predicts 4.07 mm/d precipitation, slightly larger than the observed 3.50 mm/d; however, all DYAMOND simulations underestimate the cloud cover. The diurnal precipitation cycle shows a similar amplitude to the observations; however, SAM predicts an early peak at 1500 UTC compared to observation. Precipitation has a very non-Gaussian probability distribution (Fig. 3 *B* and *D*) even when we exclude all nonprecipitating pixels from data.

The Phase 2 Intercomparison project simulated forty days during the northern-hemisphere wintertime. Forty days only partially covers the large atmospheric variability one can find in observations. For instance, this period is too short for Madden–Julian oscillation (MJO) to form, and it includes only one El Niño-Southern Oscillation (ENSO) condition. Yet, a duration of 40 d is adequate for the purpose of this work, which is to explore whether subgrid-scale structure matters for precipitation.

From 40 d simulated by SAM-DYAMOND, we discard the initial 10 d as model spin-up (34) and randomly select 10 d from the last 30 d for training, so that we capture the variability modeled by SAM, while keeping the size of the data manageable.

### Preprocessing.

To prepare the data for our neural network, we select the tropical band (20°S - 20°N) and 10 d of simulation. We discard land and retain only ocean for our training because we look for a simple model and adding land requires more input features and likely a larger dataset. The spatial resolution of SAM-DYAMOND is about 4 km, and the temporal resolution of 2D outputs is 15 min. We coarse-grain these data horizontally to subdomains that are equivalent/comparable to a GCM-size grid (e.g., ∼100 km) as follows:

[2] $$\begin{array}{c} \bar{X}\left(i,j,k\right)=\frac{1}{L^{2}}\sum_{l=L(i-1)+1}^{l=Li}\sum_{m=L(j-1)+1}^{m=Lj}X\left(l,m,k\right), \end{array}$$

where *X* is the field to be coarse-grained, *L* is the averaging (coarse-graining) factor, and *i* and *j* are the indices in the *x* and y directions. We test several coarse-graining factors from *L* = 16 to *L* = 48 (equivalently 70 × 70 km^2^ to 200 × 200 km^2^). The results displayed in the paper are based on *L* = 32, equivalent to 130 km, while the impact of *L* on the results is discussed when relevant and reported in *SI Appendix*, Table S2.

The input to the encoder of Org-NN is the high-resolution (not coarse-grained) PW anomalies with the shape of LxL (e.g., 32 × 32 for L= 32), and the anomaly is with respect to the horizontal mean over the LxL subdomain. By subtracting the mean, we separate the impact of large-scale PW from the subgrid-scale structure captured by *org*. One can alternatively scale the high-resolution PW fields by removing the mean and dividing by its SD. However, we find that this latter method reduces the models’ performance, as discussed in *SI Appendix*.

To provide the train, validation, and test datasets, we split the 10 d into 6, 2, and 2 d for train, validation, and test, respectively. Furthermore, we keep only the samples with precipitation larger than a threshold (0.05 mm/h) so that we concentrate only on predicting precipitation intensity rather than on precipitation triggering. The total number of samples is on the order of 10^8^.

### Neural Network Architecture.

We use two neural networks: a feed-forward network that is informed with only coarse-scale variables, which we refer to as the baseline-NN. We use a second architecture, which combines a feed-forward neural network with an autoencoder. The autoencoder extracts organization information from the high-resolution PW anomaly field. Fig. 2 shows these two networks. Both networks are implemented using the Tensorflow library version 2.9 (35), and the hyperparameters are tuned using the Sherpa hyperparameter tuning library (36).

#### Baseline-NN.

The baseline-NN (Fig. 2*A*) is a fully connected feed-forward neural network with 4 hidden layers and [256,256,128,64] neurons. The learning rate is scheduled to decrease with epoch and initialized to 10^−4^. The baseline-NN has access to only large-scale variables and predicts precipitation. We run four tests in which the choice of input of the baseline-NN differs and is as follows:

1. [PW, SST]
2. [PW, SST, specific humidity at 2m, temperature at 2 m]
3. [PW, SST, specific humidity at 2m, temperature at 2 m, sensible heat flux, latent heat flux]
4. [PW, SST, specific humidity at 2m, temperature at 2 m, total cloud cover]
5. [PW, SST, specific humidity at 2m, temperature at 2 m, CAPE, CIN].

The baseline-NN predicts a precipitation value for each given set of large-scale inputs.

#### Org-NN.

The architecture of Org-NN is shown in Fig. 2*B*. The encoder part of the autoencoder includes three one-dimensional convolutional layers followed by two fully connected layers. The input to this network is high-resolution PW anomalies with dimension 32 × 32 grid points (or 130 × 130 km^2^). The encoder output is *org* variables. The dimension of *org* is a hyperparameter of the network that we set to 4. The decoder has the inverse structure of the encoder. It receives *org* variables and reconstructs the original high-resolution field (e.g., 32 × 32 grid points). The NN part of this network is similar to the baseline-NN except that the latent variables, *org*, has been added as an extra input feature. The input to NN is [PW, SST, specific humidity at 2m, temperature at 2m, *org*].

*SI Appendix*, Table S2 and Fig. S5 summarizes how the choice of resolution (e.g., 32 × 32, 16 × 16, etc) and/or the dimension of *org* affects the results. Changing the dimension of *org* to 2 does not affect the prediction of precipitation; however, it reduces the reconstruction accuracy.

### Loss Function.

The baseline-NN predicts precipitation and using coarse-scale variables, while the Org-NN also reconstructs the high-resolution PW field and predicts precipitation with this extra information provided by *org*. For predicting precipitation, we use mean square error (MSE) as the loss function for the feedforward neural networks in both the baseline-NN and Org-NN models:

[3] $$\begin{array}{c} loss=\frac{1}{N}\sum_{i=0}^{N}{\left({\bar{P_{i}}}^{t}-{\bar{P_{i}}}^{p}\right)}^{2}, \end{array}$$

where N is the number of samples, $\bar{P_{t}}$ represents the coarse-grained precipitation from the DYAMOND dataset, and $\bar{P_{p}}$ represents the precipitation predicted by the neural network. MSE gives the samples with extreme precipitation greater importance than the samples with small precipitation, as these extremal values significantly increase the loss. One can alternatively use mean absolute error (MAE) as a loss function; however, we find that MAE results in a slight underestimation of extremes, while not improving the overall performance of the model.

For the baseline-NN, the above function already represents its final loss; while for Org-NN, we apply the MSE loss for the autoencoder as well and then assign weights to the loss of AE and to the loss of the feedforward NN and sum them up as the final loss function. The emphasis of our work is on predicting precipitation and extracting *org* variables but not accurately reconstructing the high-resolution two-dimensional fields. Thus, we give the MSE of the autoencoder a smaller weight (0.2) so that org-NN is more concentrated on its principal task.

An organization metric should not be sensitive to the orientation of clouds. We enforce this criterion in the autoencoder so that the *org* metric is rotationally invariant. To do so, for each high-resolution PW input of the encoder–decoder, the network applies a random rotation of 90 degrees to the input, and the encoder maps both nonrotated and randomly rotated input to the latent space. To make the org rotation-invariant, we force the encoder to map both inputs (nonrotated and rotated) to the same subspace in the latent space by adding the following term to the loss function of AE:

$$\begin{array}{cc} X^{r} & =R(X)\\ org & =e(X)\\ org^{r} & =e(X^{r})\\ loss_{\mathrm{RI}} & =\sum_{i=1}^{N}{\left(org_{i}-org_{i}^{r}\right)}^{2}/\sum_{i=1}^{N}\left|org_{i}\right|, \end{array}$$

where *R* is a rotation operator that rotates the input *X* by *nπ*/2 and *n* is random integer number. *X* is the nonrotated high-resolution PW anomaly input of the autoencoder, *X*^*r*^ is the rotated input, *e* is the encoder network, *org* is the latent representation of *X*, and *org*^*r*^ is the latent representation of *X*^*r*^. *loss*_*RI*_ is then the term we add to the loss function to make it rotation-invariant. We normalize the RI loss by dividing by the L1 norm of org values. This scaling prevents the model from minimizing the *loss*_*RI*_ by assigning small values to org rather than enforcing the rotation invariant.

The loss of Org-NN, including all three terms, is then

[4] $$\begin{array}{cc} Loss_{org-NN}= & \;\lambda_{1}\frac{1}{N}\sum_{i=0}^{N}{\left({\bar{P_{i}}}^{t}-{\bar{P_{i}}}^{p}\right)}^{2}+\lambda_{2}\frac{1}{N}\sum_{i=1}^{N}{\left(pw_{i}^{t}-pw_{i}^{p}\right)}^{2}\\ & +\lambda_{3}\frac{1}{N}\sum_{i=1}^{N}{\left(org_{i}-org_{i}^{r}\right)}^{2}/\sum_{i=1}^{N}\left|org_{i}\right|, \end{array}$$

where *P* represents coarse-scale precipitation, *pw* is the high-resolution precipitable water (e.g., 32 × 32), and N is the number of samples. The first term in Eq. **4** is the MSE loss of precipitation prediction, the second term is the MSE loss of *pw* reconstruction, and the third term is the rotation-invariant loss. *λ*_1_, *λ*_2_, and *λ*_3_ are hyperparameters and are set to 0.4, 0.2, and 0.4, respectively.

### Training and Validation Procedure.

The input to each network is in the form of minibatches so that we do not train on individual samples one by one, but rather on an ensemble of samples. Thus, the shape of inputs to the baseline-NN and NN part of Org-NN is [n_*batch*_,*n*_*f*_], where *n*_*batch*_ is the number of samples randomly selected in each minibatch and *n*_*f*_ is the number of fields that we pass as input. For example, for the baseline-NN where we pass PW and SST as input, *n*_*f*_ equals 2. The shape of the input for the AE is [n_*batch*_,L_*x*_,L_*x*_,1], and L_*x*_ is the dimension of the high-resolution field. We examine our model on 4 resolutions: 200 km, 130 km, 100 km, and 75 km that correspond to L_*x*_ = 48, 32, 24, and 16, respectively. The model is trained on the minibatches of 128 samples, for 100 epochs. In order to prevent overfitting, we implement the strategy of early stopping. At each iteration, the network computes the loss averaged over samples in one minibatch. This loss value is backpropagated through the network, and its derivative with respect to each neural network’s parameter is computed. The neural network’s parameters are then updated using the ADAM algorithm. This process is repeated over all minibatches, which corresponds to one epoch. At the end of each epoch, the network’s performance is validated using the validation dataset, which the network has not seen while being trained. The training-validation process continues until either the total number of epochs is reached or until the early stopping criteria are met. Here, the early stopping has patience equal to 10 epochs. This means that if the validation loss does not improve for five consecutive epochs, the network training stops. Early stopping was used to prevent the network’s overfitting so that the model can have a better generalization ability.

### Evaluation Metric.

To evaluate the neural network’s performance, we use *R*^2^, a commonly used metric for quantifying the performance of regression models.

Let us say we have a dataset of n values *y*_1_, *y*_2_, …, *y*_*n*_, each associated with a predicted value *p*_1_, *p*_2_, …, *p*_*n*_. *R*^2^ of these data is then defined as

$$R^{2}=1-\frac{\sum_{i=1}^{i=n}{\left(y_{i}-p_{i}\right)}^{2}}{\sum_{i=1}^{i=n}\left(y_{i}-\bar{y}\right)},$$

where $\bar{y}$ is the true mean. The numerator measures the residual of the prediction, while the denominator measures the variance of the true data. A perfect prediction with zero residual has *R*^2^ = 1. A model that assigns mean value, $\bar{y}$, to each *p*_*i*_ has *R*^2^ = 0.

Given the spatiotemporal variability of precipitation, one can compute *R*^2^ in several manners. Let us say P = P(time, lat,lon), then we can compute *R*^2^:

1. By expanding P and creating a large list, then computing an overall *R*^2^: This score has the lowest value.
2. By computing *R*^2^ across one of the dimensions of the data: For example, computing *R*^2^ for time series of precipitation at each (lat, lon). Fig. 3 *C* and *F* are computed following this approach. Alternatively, one can bin precipitation based on PW and then compute *R*^2^ of precipitation over each bin of PW (green curves in Fig. 3 *A* and *D*).
3. By first averaging precipitation across one or two dimensions and then computing *R*^2^ of the mean profile: For instance, one can first average true and predicted precipitation zonally and temporally and then compute *R*^2^ of the averaged profiles. Alternatively, one can first average precipitation over bins of PW and then compute *R*^2^ for averaged precipitation.

In this work, we employ the first and second methods to compute *R*^2^. The first method gives *R*^2^ ∼ 0.45 for baseline and 0.9 for Org-NN. The second method is used in Fig. 3*C* as well as Fig. 3*B*, green line.

We have not computed *R*^2^ based on the third method as it overestimates the models’ performance. One can imagine a NN that assigns the zonally averaged precipitation to all longitude points without predicting any longitudinal variability. Following the third approach mentioned above and computing *R*^2^ of zonally averaged precipitation would evidently overestimate the performance of the imagined NN. The same will happen if we compute *R*^2^ using precipitation averaged over bins of PW. Thus, although the averaged predicted precipitation over bins of PW closely follows the true profile (Fig. 3*A*), the baseline model has a low skill (*R*^2^ < 0.5) as revealed by Fig. 3*A* green line.

## Supplementary Material

Appendix 01 (PDF)

## Data Availability

[1) Simulation of atmosphere 2) codes for preprocessing the data and training the neural networks]. Data have been deposited in [1) DKRZ 2) Github] 1) https://www.esiwace.eu/services/dyamond-initiative 2) https://github.com/Sshamekh/Precip-org) (37).
