# Supplementary material for: Implicit learning of convective organization explains precipitation stochasticity
Source: Proc Natl Acad Sci U S A. 2023 May 8;120(20):e2216158120. doi: 10.1073/pnas.2216158120 (PMC10193982; doi:10.1073/pnas.2216158120)
Supplement: Supplementary file 1 — Appendix 01 (PDF) [file pnas.2216158120.sapp.pdf]

Supporting Information for

Implicit learning of convective organization explains precipitation stochasticity

S. Shamekh, K. D. Lamb, Y. Huang, P. Gentine

S. Shamekh.
E-mail: ss6287@columbia.edu

This PDF file includes:

Figs. S1 to S13
Tables S1 to S2
SI References

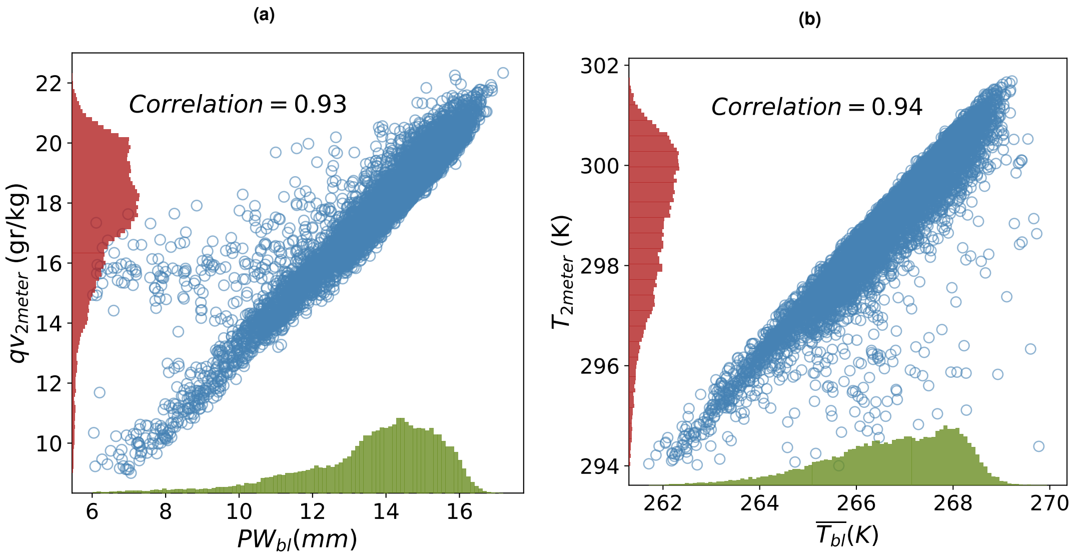

Fig. S1. The plot on the left shows the joint distribution of boundary layer precipitable water (PW_{bl}) and near surface humidity (qv_{2m}). The green and red histograms are respectively the marginal distribution of PW_{bl} and qv_{2m}. The plot on the right shows the same but for the averaged boundary layer temperature ($\overline{T_b l}$) and temperature at 2 meters ($T2meters$). Boundary layer is defined to be above 850 hPa.

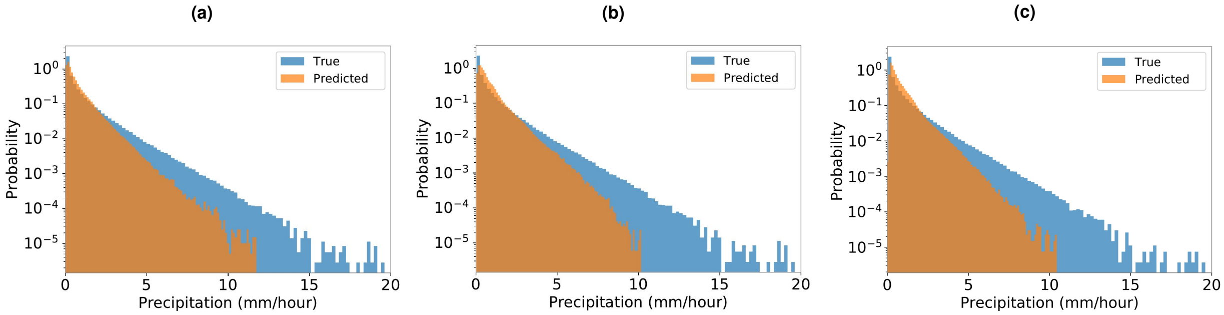

Fig. S2. The plots show the PDF of true (blue) and predicted (orange) precipitation for different choices of inputs to the Baseline-NN. The inputs are a) [PW, SST, specific humidity at 2m, temperature at 2 m, sensible heat flux, latent heat flux], b) [PW, SST, specific humidity at 2m, temperature at 2 m, total cloud cover], and c) [PW, SST, specific humidity at 2m, temperature at 2 m, CAPE, CIN].

Supplementary tests

Scaling high-resolution PW. We investigate scaling each high-resolution PW anomaly by its standard deviation before passing to Org-NN. Removing the first and second moment of PW from the input reduces the performance of Org-NN to some extent, especially the prediction of extremes (Figure S13), indicating the importance of the second moment for the prediction. To verify the informativity of the second moment, we run a test where we replace the *org* learned by the auto-encoder with the standard deviation of the high-resolution PW (hereafter std_{PW}). We find that the Org-NN informed by std_{PW} performs better than Baseline-NN but worse than org-NN, which learns *org* directly. This result indicates that the sub-grid scale moisture distribution (the second moment of PW) encodes some relevant information for predicting precipitation.

A. Including land in the input. The paper's main focus was on the predictability of precipitation over the ocean, so the land has been masked out. Previous studies(1) find that a neural network trained on both land and ocean does not accurately predict both groups and tends to be more skillful over land with more substantial variability. Here, we investigate precipitation predictability when Org-NN is trained on both land and ocean, thus inducing tropical land alongside the ocean in the input of Org-NN. Figure S12 shows that Org-NN can predict precipitation over both land and ocean. However, the R^2 of this prediction is 0.82, slightly smaller than training on only ocean data. Despite a small fraction of very intense precipitation (> 21 mm/hr), the model accurately reproduces the probability distribution of precipitation and precipitation extremes. These very intense precipitation events are tropical land precipitation, as they are not present in Figure 3.d where we masked out the land from the data. This finding shows that when *org* is known, precipitation over both land and ocean can be accurately predicted from a small set of two-dimensional inputs (surface temperature, PW, temperature at 2 meters, and specific humidity at 2 meters).

Table S1. **Description of organization metrics used in Figure S3. Detailed definitions are provided in Janssens et al. (2021)**

Category	Name	Description
Statistical properties	PR	precipitation rate
	Ncloud	cloud object number
	CLF	cloud occurrence frequency
	PWmean	mean precipitable water
	PWstd	standard deviation of precipitable water
	CLWvarr	cloud liquid water variance ratio
Object-based metrics	OSmean	mean rectangular contiguous clear-sky area
	lmean	mean cloud object size
	lmax	max cloud object size
	IorgP	organization index
	SCAI	Simple Convective Aggregation Index
	COP	Convective Organisation Potential
	RDFmax	maximum value of the cloud object radial distribution function
Scale decomposition metrics	SizeExp	exponent of cloud size distribution (power law fit)
	FracDim	box-counting dimension of cloud boundaries
	SpecL	spectral length scale

Table S2. The impact of resolution and org dimension on the prediction of precipitation by the Org-NN model

Resolution (km^2)	grids	org size	R^2
70x70	16x16	4	0.82
100x100	24x24	4	0.82
100x100	24x24	2	0.80
135x135	32x32	4	0.81
135x135	32x32	2	0.79
200x200	48x48	4	0.78

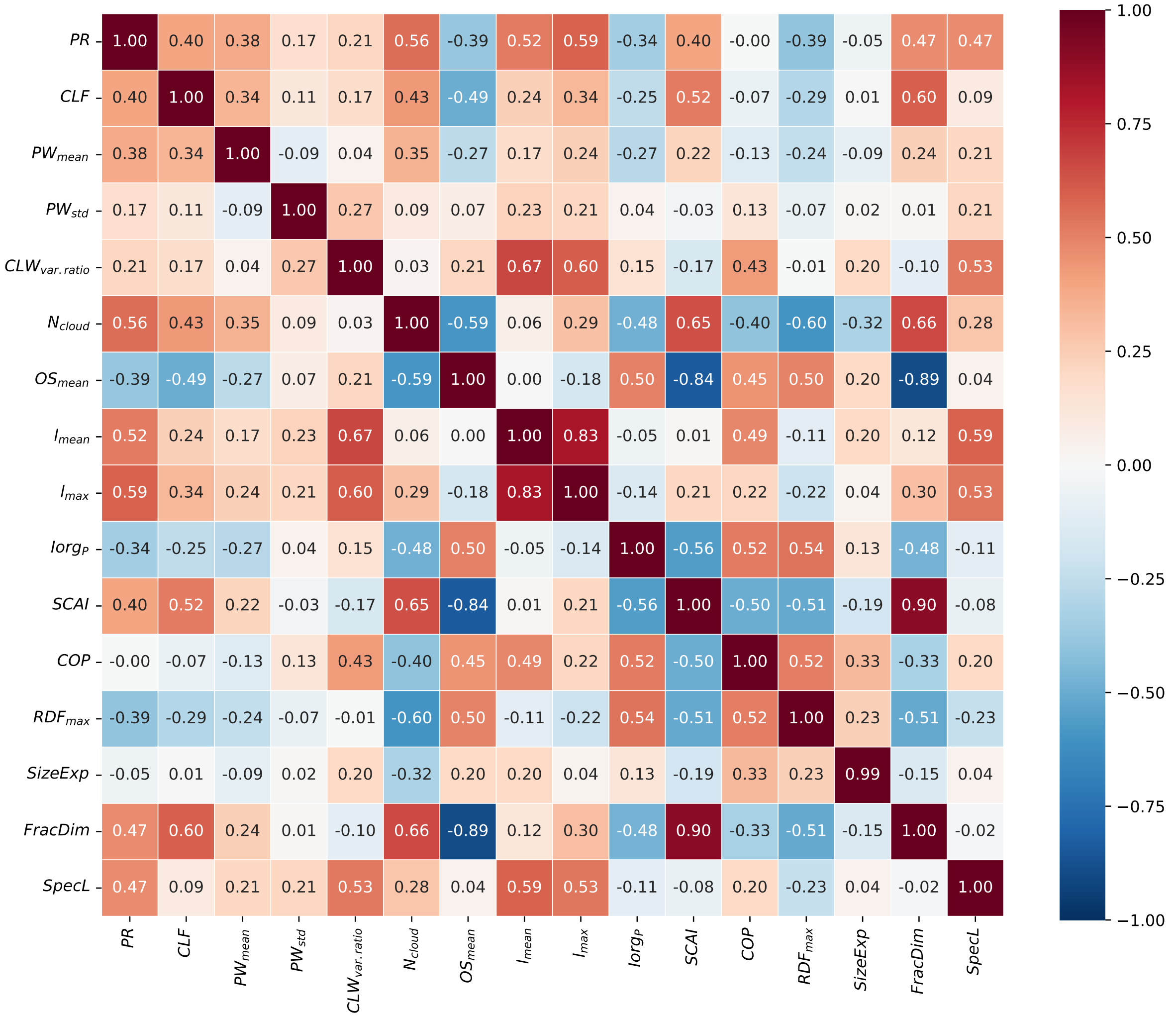

Fig. S3. The heat-map shows pairwise correlations between pairs of previously defined organization metrics,collected by Janssens et al. (2021)(2), applied to SAM data. Simple descriptions for the metrics are provided with Table S1. Organization metrics have been measured for domains of 10x10 degree every 3 hours.

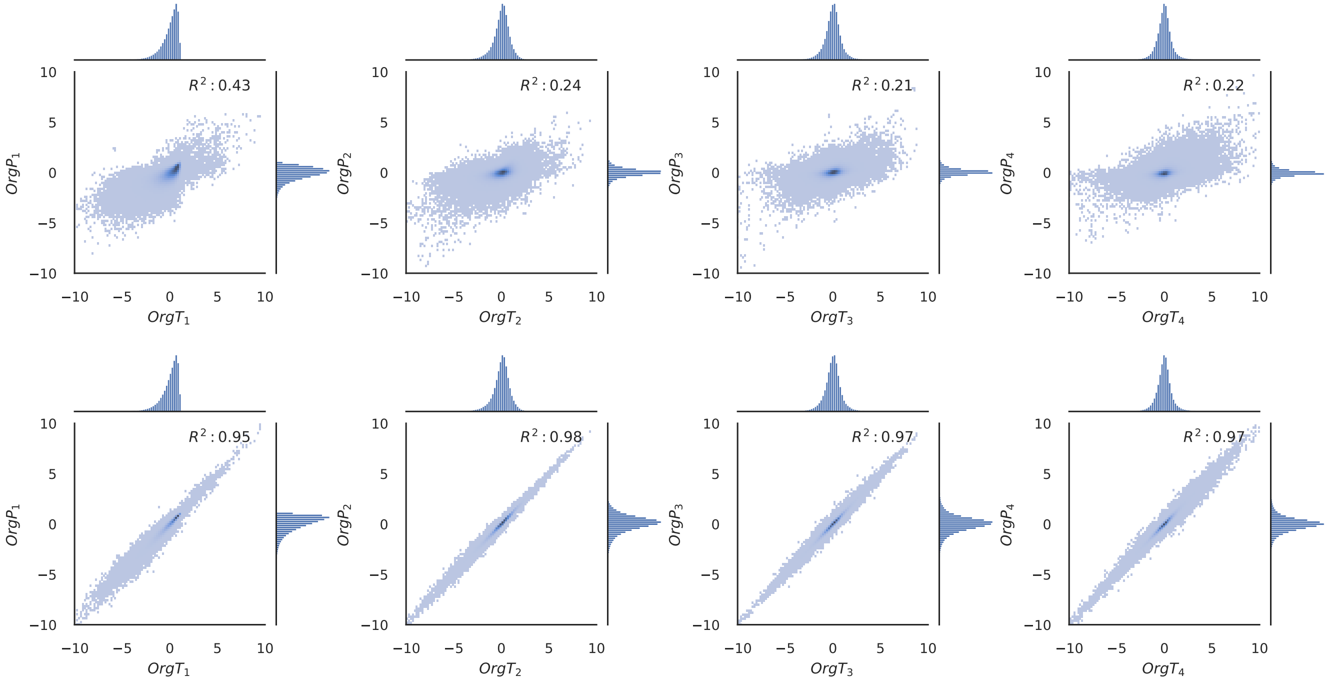

Fig. S4. The plots on first row show the prediction of the 4 org variables using a neural network that receives large scale variables at the current and two previous time steps (n_{LS} = 4) (Equation 1 withn_{LS}= 4 and n_{org} = 0). The subscript P denotes the predictions by simple neural network and T denotes the true values extracted from the auto-encoder. The histograms show the marginal distribution of true (at top) and predicted (at right side) org. The plots on the second row shows the same as first row but for n_{LS}= 2 and n_{org} = 1.

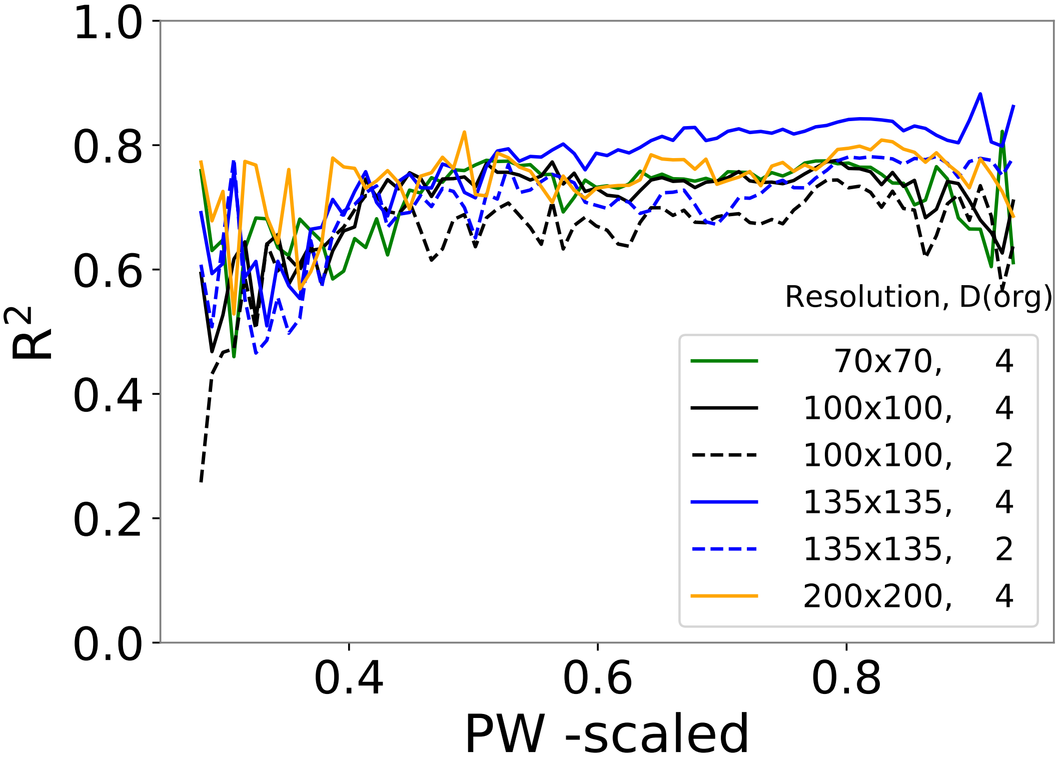

Fig. S5. Panel shows the R^2 across PW bins for org-NN prediction trained on different resolutions of the inputs and org dimension.

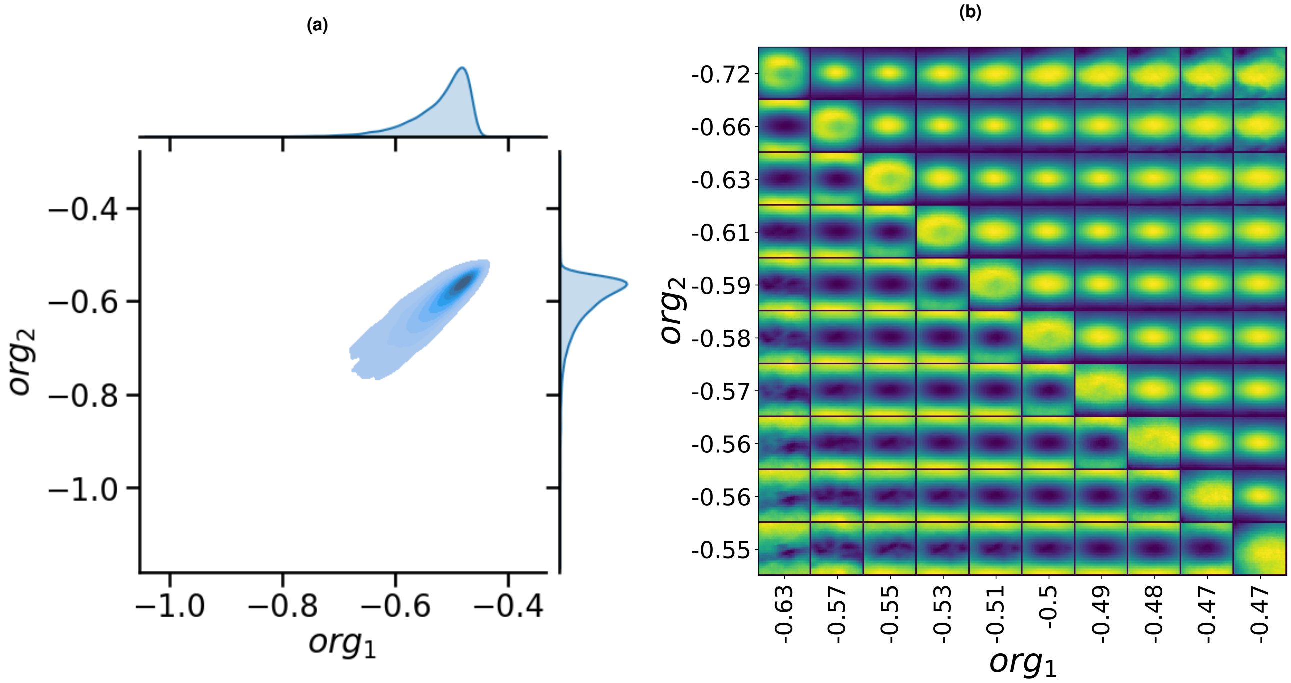

Fig. S6. Latent space of org a) Joint probability distribution of org_1 and org_2 for the $d_{org} = 2$ case from predictions of the encoder trained on the DYAMOND simulations. The histograms of each distribution are shown on the top (for org_1) and right side of the plot (for org_2). b) Visualization of the latent representation for the $d_{org} = 2$ case. Color scaling is relative to the minimum and maximum values for PW in each reconstructed high resolution field to best show small scale contrast. Values for the org_1 and org_2 are chosen to be the midpoint of the deciles of the observed distributions.

References

1. G Mooers, et al., Assessing the potential of deep learning for emulating cloud superparameterization in climate models with real-geography boundary conditions. *J. Adv. Model. Earth Syst.* **13**, e2020MS002385 (2021) e2020MS002385 2020MS002385.
2. M Janssens, et al., Cloud patterns in the trades have four interpretable dimensions. *Geophys. Res. Lett.* **48**, e2020GL091001 (2021) e2020GL091001 2020GL091001.

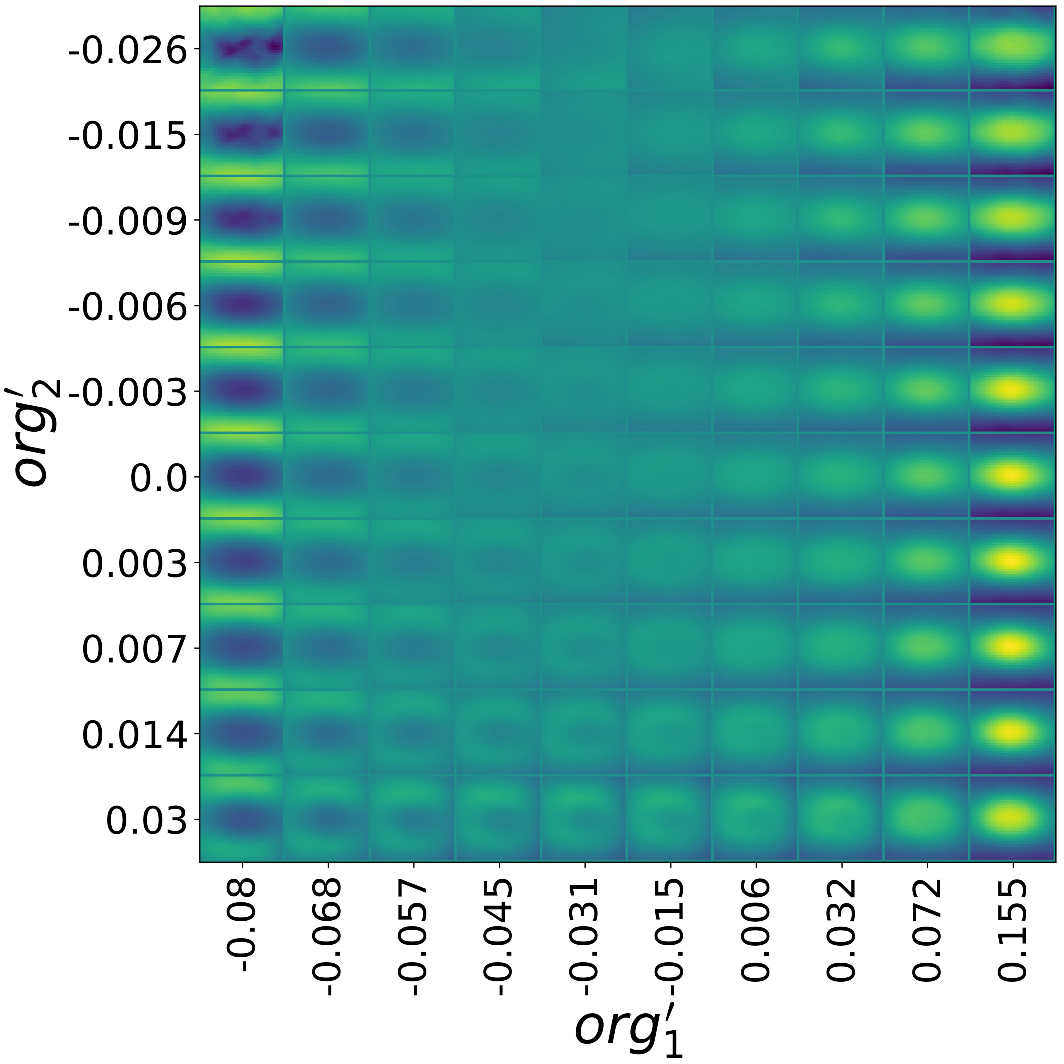

Fig. S7. As in Figure 5, but color scaling for PW is relative to the maximum and minimum across all the reconstructed high resolution fields, rather than each individual field.

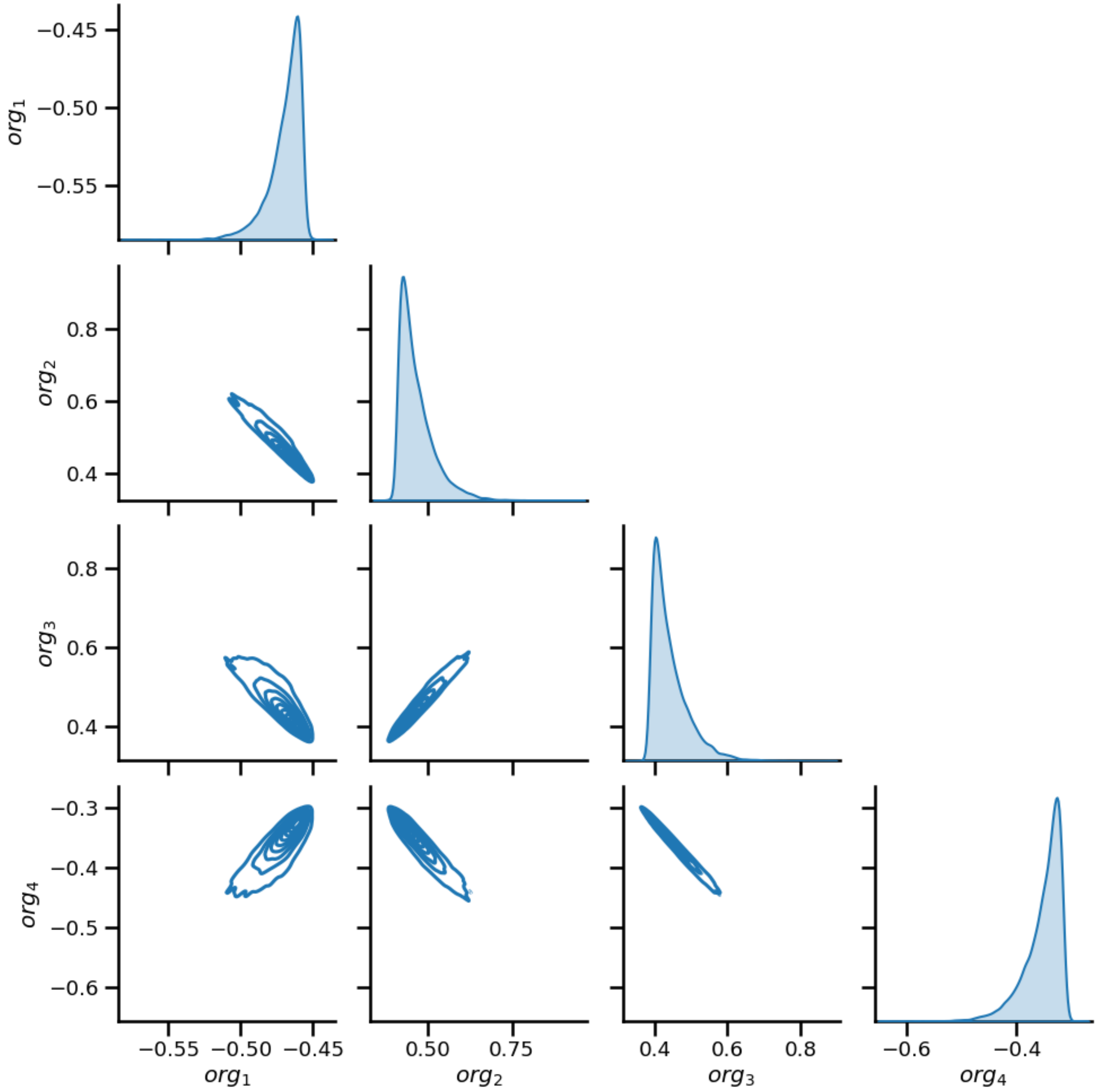

Fig. S8. Marginal distributions and joint probability distributions of org parameters from the DYAMOND simulations for the $d_{org} = 4$ case.

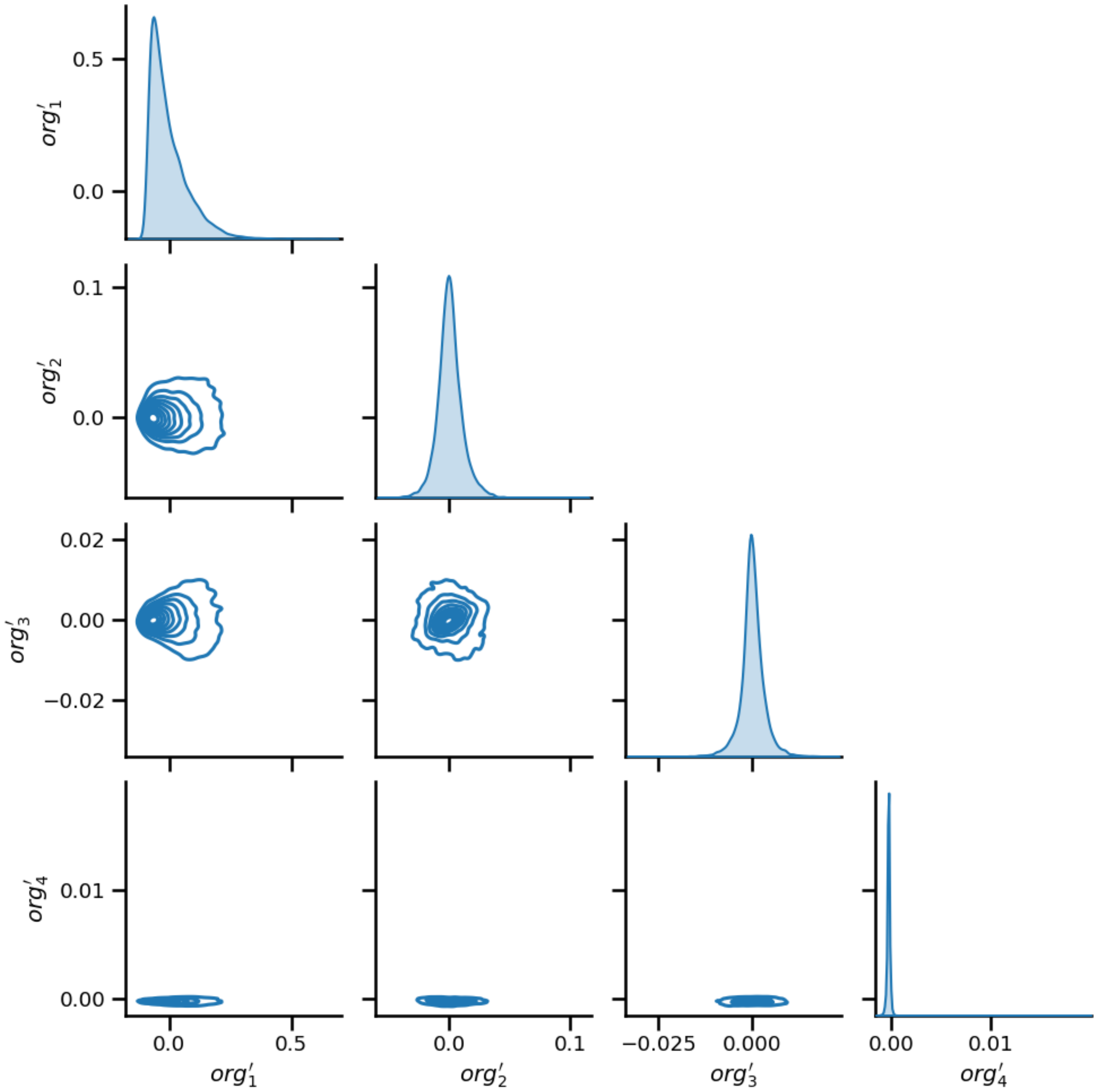

Fig. S9. Marginal distributions and joint probability distributions of the first 4 principal components of the org parameters (denoted as org') from the DYAMOND simulations for the $d_{org} = 4$ case.

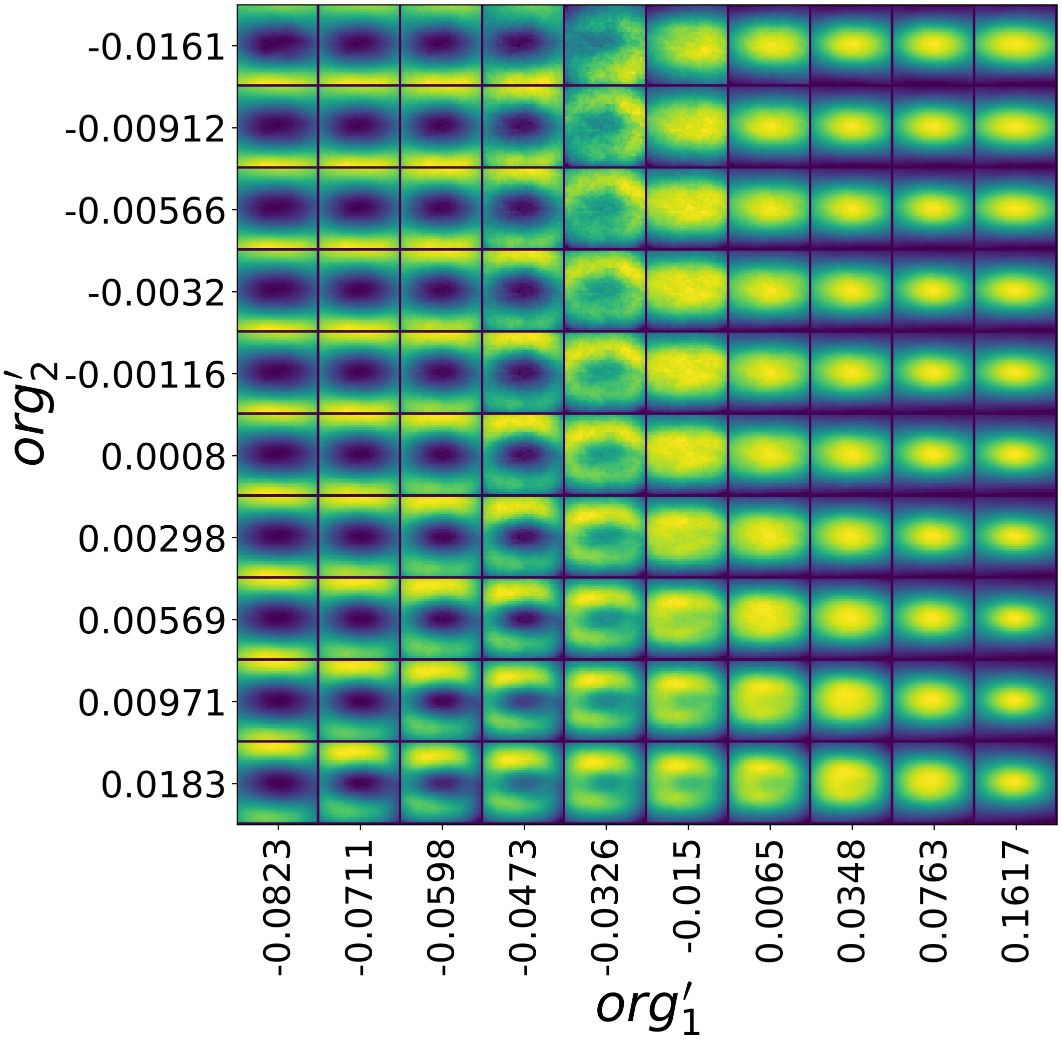

Fig. S10. Visualization of the latent space for the first two principal components for the $d_{org} = 4$ case. Each subplot shows the reconstructed high resolution fields of PW across the distribution of values for org'_1 and org'_2, with the values for org'_3 and org'_4 held fixed (to the center values of their distributions). Values for the org'_1 and org'_2 are chosen to be the midpoint of the deciles of the observed distributions.

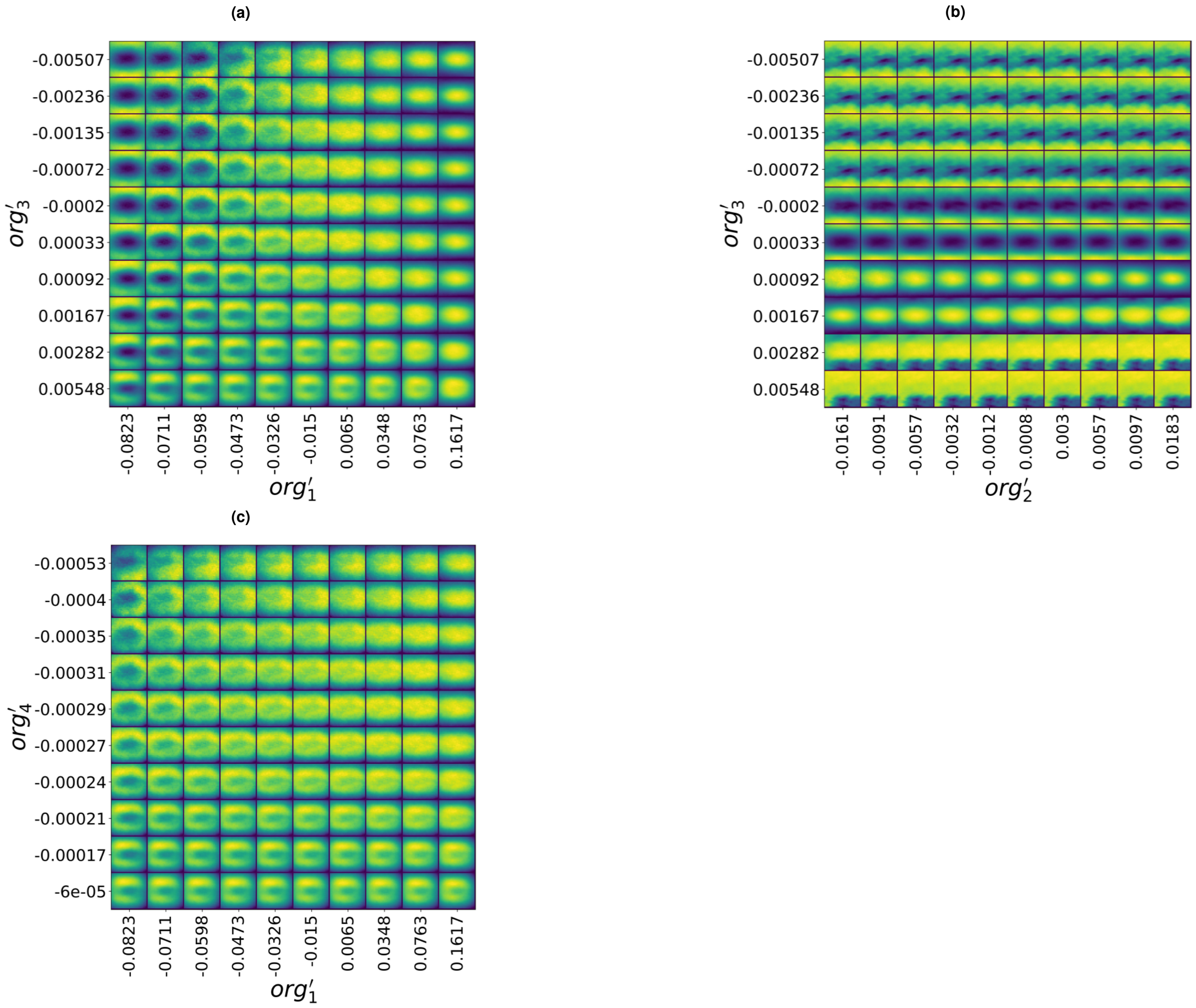

Fig. S11. Latent space of org for $d_{org} = 4$ a) Visualization of the latent space for the first and third principal components for the $d_{org} = 4$ case. b) for the second and third principal components. c) for the first and fourth principal components.

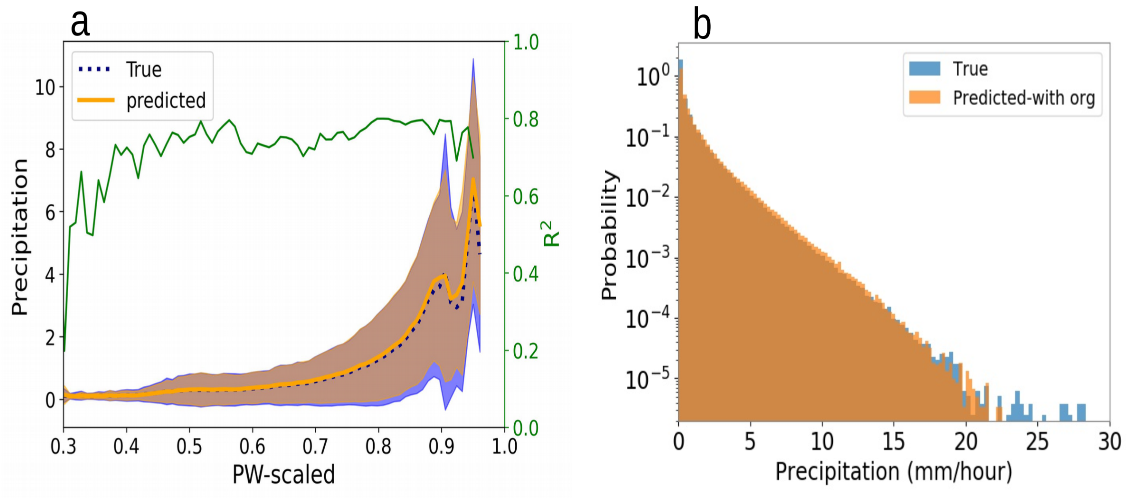

Fig. S12. The plot shows the performance of Org-NN trained on both land and ocean. The large-scale inputs (surface temperature, PW, qv2m, and T2m) and precipitation are coarse-grained by averaging over a block of 32x32 grids (130x130km^2), and the dimension of org is set to 4. Panel (a) shows true (blue) and predicted (orange) coarse-scale precipitation averaged over PW bins (1 mm). Shading indicates the standard deviation of coarse-scale precipitation for each bin of PW. The green line plots the R^2 across PW bins. Panel b shows the probability density function (pdf) of precipitation for true (blue) and prediction from panel a (orange).

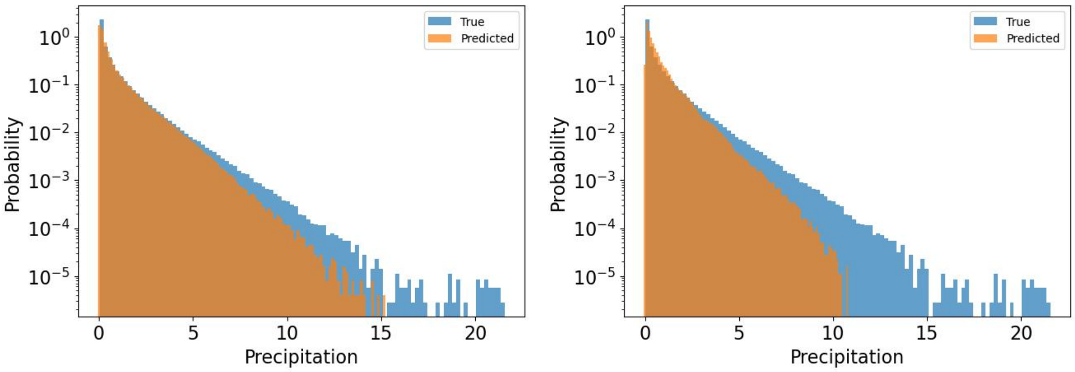

Fig. S13. Org-NN trained with scaled 2D-PW, R2 = 0.55 for the right panel and 0.70 for the left
